# Temporal Pattern and Clinical Value of Serum GFAP in Acute Ischemic Stroke: Results from Two Prospective German Cohorts

**DOI:** 10.1007/s12975-026-01458-1

**Published:** 2026-06-15

**Authors:** Lorenzo Barba, Christoph Vollmuth, Patrick Oeckl, Steffen Halbgebauer, Christian Hametner, Fabian Essig, Peter U. Heuschmann, Alexander M. Kollikowski, Mirko Pham, Michael K. Schuhmann, Petra Steinacker, Yashar Bahramsari, Annemarie Thaele, Caroline Kulitze, Samir Abu-Rumeileh, Karl Georg Haeusler, Guido Stoll, Hermann Neugebauer, Markus Otto

**Affiliations:** 1 Department of Neurology, Martin-Luther-University of Halle-Wittenberg, Halle (Saale), Germany; 2 Department of Neurology, University Hospital Würzburg, Würzburg, Germany; 3 Department of Neurology, University of Ulm, Ulm, Germany; 4 German Center for Neurodegenerative Diseases (DZNE e.V.), Ulm, Germany; 5 Institute for Clinical Epidemiology and Biometry, University of Würzburg, Würzburg, Germany; 6 Institute for Medical Data Science, University Hospital Würzburg, Würzburg, Germany; 7 Department of Neuroradiology, University Hospital Würzburg, Würzburg, Germany; 8 Clinic II for Internal Medicine, Asklepios Clinic Nord-Heidberg, Hamburg, Germany; 9 Institute of Experimental Biomedicine I, University Hospital Würzburg, Würzburg, Germany

## Abstract

Serum glial fibrillary acidic protein (sGFAP) is an astroglial biomarker preliminarily investigated in acute ischemic stroke (AIS). We aimed to investigate its temporal pattern and clinical value in two German AIS cohorts. Using immunoassay, we measured sGFAP concentrations in 952 samples of 572 AIS patient and 32 samples of controls recruited in Halle [AIS *n* = 102, age: 70.9 (± 15.9) years, 52.0% males; controls *n* = 32, age: 47.6 (± 18.8) years, 59.4% males] and Würzburg [AIS *n* = 470, age 74.6 (± 12.8) years, 48.3% males]. In AIS patients, we stratified samples according to the time from symptom onset to blood sampling, namely at D1 (< 24 h), D2 (24–48 h), D3 (48–72 h) and D5-7 (96–144 h), and tested associations between biomarker levels and clinical data. sGFAP level was increased at all timepoints after AIS compared to control subjects (*p* < 0.001, age-adjusted *p* < 0.01) with significant changes over time (D1 < D2< D3 ≥ D5-7). We found increased sGFAP concentrations in AIS patients who had altered mental status on admission (*p* < 0.01), hemorrhagic transformation of AIS (HT, *p* < 0.001) as well as systemic infections such as pneumonia (*p* < 0.001). Higher D1 sGFAP concentrations were associated with higher likelihood of modified Rankin Scale > 2 at 3 months [aOR = 1.015 (95%CI = 1.005–1.025), *p* = 0.004] and a shorter survival at longest follow-up (median: 576 days, max 922 days) [highest tertile vs. lowest tertile HR = 2.94 (95%CI = 1.21–7.17), *p* = 0.018]. sGFAP is elevated after AIS and of informative value regarding the risk HT and infections, as well as on the clinical outcome at follow-up.

**Supplementary Information:**

The online version contains supplementary material available at 10.1007/s12975-026-01458-1.

## Introduction

Blood-based biomarkers reflecting the severity of brain injury are being intensively investigated for improving prognostic evaluation of patients affected by acute ischemic stroke (AIS). Glial fibrillary acidic protein (GFAP) is a candidate biomarker of astroglial injury whose blood level is increased in several neurological disorders including AIS [1]. Previous studies reported on the rapid increase of blood GFAP concentrations in the first hours after IS onset with dynamic changes over time [2–5]. The assessment of serum GFAP (sGFAP), either alone or in combination with other markers of neuronal injury such as serum neurofilament light chain (sNfL), improves the prognostic confidence for clinical outcomes at 3 months after AIS (i.e., functional disability and death) in comparison to standard clinical and radiological evaluation [6–8]. Moreover, GFAP seems to be a sensitive marker of brain hemorrhage which could help in the discrimination of hemorrhagic vs. ischemic stroke in pre-hospital settings [9] as well as for hemorrhagic transformation (HT) of AIS [10].

Despite the encouraging findings, several issues limit the generalizability of previous results and, thus, the clinical implementation of the biomarker in stroke medicine. First, results on blood GFAP level over time were reported only in single-center cohorts with relatively small sample size or with incomplete data on serial sampling [2, 3]. Second, the magnitude of biomarker level increase compared to control subjects remains uncertain given that most AIS cohorts were published without corresponding control group [2, 9]. Third, there is a lack of data on sGFAP in some stroke subtypes, such as AIS of the posterior circulation due to basilar artery (BA) occlusion, as well as in association with acute treatment outcomes. Fourth, despite the frequent occurrence of infections as systemic complications of AIS, their associations with biomarkers such as sGFAP and sNfL remain unknown. Fifth, the prognostic value of sGFAP for clinical outcomes after 3 months from AIS independently from HT is unclear [2, 3].

To address these issues and to better understand the clinical usefulness of sGFAP for AIS management, we conducted a comprehensive evaluation of sGFAP concentrations in AIS patients recruited in two prospective AIS cohorts. We collected serial samples at different timepoints during the first week after AIS, explored the associations between sGFAP and AIS characteristics and analyzed its value, in comparison to that of sNfL, for predicting short- and middle-term clinical outcomes after AIS.

## Methods

### Study Population

We included in this study a total of 952 serum samples with available biomarker data collected in two German recruiting stroke centers: 383 serum samples of 102 AIS patients recruited between June 2023 and October 2024 and 32 samples of control subjects enrolled at the Department of Neurology, Martin-Luther-University Halle-Wittenberg (Halle Saale, Germany, total *n* = 415 samples) and 537 samples of 470 AIS patients recruited at the Department of Neurology, University of Würzburg (Würzburg, Germany). Inclusion criteria of the Halle cohort were: (1) diagnosis of AIS; (2) hospital admission and first blood sampling within 24 h from known symptom onset; (3) modified Rankin Scale (mRS) 3 months after stroke. The control group was recruited in 2023–2024 and included subjects affected by non-cerebrovascular and non-inflammatory neurological diseases (idiopathic cranial nerve palsy *n* = 6, headache *n* = 11, polyneuropathy *n* = 1, psychogenic non-epileptic seizure *n* = 2, vertigo *n* = 1) as well as healthy controls (*n* = 12) with available samples for all measurements. Patients of the Würzburg cohort were enrolled in a prospective observational study between June 2020 and September 2022 at the Department of Neurology of the University of Würzburg (registry number: DRKS00022064). In the Würzburg cohort, adult patients with AIS were included if they had a National Institute of Health Stroke Scale (NIHSS) score at hospital admission of at least 6 points or if they underwent mechanical thrombectomy (MT) for large vessel occlusion (LVO). Data on a subgroup of the Würzburg cohort have been previously published (see Supp. Figure S1 for full study protocol) [6–8]. In both cohorts, we collected detailed clinical, radiological and biochemical data during the hospitalization period, as well as data on the functional status at 90-day follow-up (collected data of both cohorts are presented in detail in **Supplementary Materials**).

### Neuroimaging Data

Detailed description of acquisition and analysis of neuroimaging data is reported in the **Supplement**. Briefly, we collected data on the Alberta Stroke Program Early CT Score (ASPECTS) [11] on native non-contrast CT upon hospital admission and after 24 h, on recanalization success after MT as modified Treatment In Cerebral Infarction (mTICI) score [12], and hemorrhagic transformation (HT) of AIS according to the Heidelberg Bleeding Classification (HBC) [13].

### Blood Sample Collection and Analysis

Serum samples were collected according to international standardized procedures and stored at −80 °C upon biomarker analysis. Biomarker measurement was performed at the Neurochemical Laboratory of Martin-Luther-University (Halle, Germany) by using commercially available immunoassays for sGFAP, namely the Simoa GFAP Discovery Kit run on a HD-X platform (Quanterix Inc., Lexington, USA), and for sNfL, i.e. the Human NF-L Simple Plex assay for the Ella microfluidic system (BioTechne, Minneapolis, USA). Internal controls were run on each plate to guarantee comparability of results. Mean coefficients of intra- and inter-assay variability were below 15% and 20% in all measurements. Samples were distinguished in different time points according to the time (in days) from clinical onset to blood sampling. Timepoints were defined as follows: D1 (day 1, < 24 h), D2 (day 2, 24–48 h), D3 (day 3, 48–72 h), D5-7 (days 5–7, 96–144 h). For a subgroup of patients from the Halle cohort (*n* = 40), we obtained two samples within 24 h from onset, namely before neuroimaging upon hospital arrival (D0 sample) and after transfer at stroke unit after neuroimaging and, if administered, acute treatment (D1 sample). For 41 patients of the Würzburg cohort, serial samples during the first week after AIS onset were collected (same temporal stratification of samples as for the Halle cohort). Numbers of patients with available biomarker data at each time point are reported in Table 1.

**Table 1:** Core demographic and clinical data of the study cohorts

	Halle		Würzburg	p*
	controls (*n* = 32)	AIS (*n* = 102)	AIS (*n* = 470)	
Age [mean (± sd)]	47.6 (± 18.8)	70.9 (± 15.9)	74.6 (± 12.8)	< 0.001
Male sex [n (%)]	19 (59.4)	53 (52.0)	227 (48.3)	0.627
NIHSS on admission	-	7 (4–15)	13 (9–17)	-
LVO [n (%)]	-	53 (52.0)	416 (88.5)	-
Etiology [n (%)]				
LAA	-	29 (28.4)	79 (16.8)	-
CE	-	35 (34.3)	213 (45.3)	-
SVD	-	10 (9.8)	0 (0)	-
other determined etiology	-	1 (1.0)	16 (3.4)	-
cryptogenic	-	26 (25.5)	147 (31.3)	-
concurrent etiology	-	1 (1.0)	15 (3.2)	-
Comorbidities				
AF (known or newly detected)	-	22 (21.6)	212 (45.1)	-
Arterial hypertension	-	79 (77.5)	315 (67.0)	-
Diabetes mellitus	-	25 (24.5)	98 (20.9)	-
Supply territory of ischemic lesion [n (%)]				
ACA	-	1 (1.0)	5 (1.1)	-
MCA	-	87 (85.3)	425 (90.4)	-
PCA	-	5 (4.9)	7 (1.5)	-
VA or BA	-	9 (8.8)	33 (7.0)	-
Revascularization therapy [n (%)]				
IVT	-	57 (55.9)	180 (38.3)	-
MT	-	47 (46.1)	359 (76.4)	-
MT + IVT	-	19 (18.6)	130 (27.7)	-
Clinical outcomes [n (%)]**				
intra-hospital mortality	-	17 (16.7)	121/477 (25.4)	-
mortality at 3 months	-	22 (21.6)	161/464 (34.7)	-
mRS of 3–6 at 3 months	-	51 (50.0)	324/464 (69.8)	-
Timepoints of blood sampling [n (%)]				
D1	32 (100.0)	102 (100.0)	134 (28.5)	-
D2	-	97 (95.1)	236 (50.2)	-
D3	-	95 (93.1)	123 (26.2)	-
D5-7	-	89 (87.3)	44 (9.4)	-
sGFAP [ng/ml]				
D1	0.13 (0.09–0.19)	1.51 (0.63–6.32)	3.11 (0.66–11.44)	< 0.001
D2	-	2.70 (0.80–11.20)	7.32 (1.66–21.98)	< 0.001
D3	-	3.20 (1.20–22.70)	9.21 (2.55–37.78)	< 0.001
D5-7	-	2.05 (0.88–12.10)	9.42 (3.26–22.96)	< 0.001
sNfL [pg/ml]				
D1	7.0 (3.3–13.4)	66.7 (29.0–139.5)	72.0 (33.3–178.5)	< 0.001
D2	-	114.0 (50.7–182.5)	107.0 (57.5–221.0)	< 0.001
D3	-	137.0 (59.5–271.0)	171.5 (81.7–330.0)	< 0.001
D5-7	-	202.0 (97.3–383.5)	546.0 (262.5–1086.0)	< 0.001

Continuous data are reported as median value (interquartile range) except for age, which was reported as mean value (± standard deviation). Comparisons were performed with Mann-Whitney U test and chi-squared tests for continuous and categorical variables, respectively. *Controls vs. AIS (Halle). **for data on clinical outcomes in the Würzburg cohort, we reported also the number of cases with available data

*ACA* anterior cerebral artery, *AF* atrial fibrillation, *AIS* acute ischemic stroke, *BA* basilar artery, *CE* cardioembolism, *IVT* intravenous thrombolysis, *LAA* large artery atheroscleris, *LVO* large vessel occlusion, *MCA* middle cerebral artery, *mRS* modified rankin scale, *MT* mechanical thrombectomy, *NIHSS* national institute of health stroke scale, *PCA* posterior cerebral artery, *sGFAP* serum glial fibrillary acidic protein, *sNfL* serum neurofilament light chain protein, *SVD* small vessel disease, *VA* vertebral artery

### Statistical Analysis

Statistical analysis was conducted on R studio v.4.2.2 (R foundation, Vienna, Austria) and GraphPad v.8 (GraphPad Software, La Jolla, USA). Comparisons of categorical and continuous variables were performed with the chi-squared test and either with the Mann-Whitney U test (for two groups) or the Kruskal-Wallis test followed by Dunn-Bonferroni’s post-hoc correction (for three or more groups). To compare repeated biomarker measurements at different timepoints, we used the Friedman test with multiple comparison by correcting for false discovery rate (FDR). Receiver operating characteristic (ROC) analysis was performed to assess the discriminative accuracy of biomarkers, and best biomarker cutoffs were found by maximizing the Youden index. Multivariable generalized linear regression models (GLMs) were built for analysing the associations of biomarker concentrations at distinct time points and multiple clinical variables. Covariables of multivariable models included (if not other specified) age and renal function as estimated glomerular filtration rate (eGFR, see **Supplement**) given their association with sGFAP and sNfL in reference healthy populations [14, 15], as well as the National Institute of Health Stroke Scale (NIHSS) score on hospital admission. In healthy subjects, female sex was associated with an increase of approximately 14% in sGFAP concentrations, whereas it had no significant influence on sNfL level [15]. In both AIS cohorts, we did not find significant difference in biomarker levels between male and female participants and, to increase analysis sensitivity, we did not include sex in multivariable models. In the Halle cohort, we used Kaplan-Meier and both univariate and multivariate Cox regression analysis to investigate the relationship between biomarker levels and risk of mortality at follow-up, which was conducted until the 6th November 2025. Missing data were not imputed. Statistical significance was set at p-values < 0.05.

## Results

### Clinical Characteristics and Available Blood Samples of AIS Patients

#### Halle Cohort

Patients had mean age of 70.9 years (± 15.9 years), 53/102 AIS patients were male (52.0%) and median NIHSS score at hospital admission was 7 points (interquartile range, IQR: 4–15). Fifty-seven of 102 patients (55.9%) underwent intravenous thrombolysis (IVT) and 47 (46.1%) underwent MT (19 patients received both therapies, 18.6%). AIS was more frequently due to large-artery atherosclerosis (LAA, 29/102, 28.4%) or cardioembolism (CE, 35/102, 34.3%) (Table 1). Number of samples available per participant was 1 in 4 patients (3.9%), 2 in 3 patients (2.9%), 3 in 7 patients (6.9%) and 4 in 88 patients (86.3%).

#### Würzburg Cohort

Patients had mean age of 74.6 years (± 12.8 years) and median admission NIHSS score of 13 points (IQR: 9–17); 227/470 patients (48.3%) were male. As a part of the inclusion criteria of this prospective cohort, MT was indicated in 359/470 (76.4%) patients, whereas IVT was administered in 180 patients (38.3%) (130, 27.7% received both therapies). In this cohort, proportion of AIS due to CE was slightly higher (45.3%) (further details in Table 1). Number of available samples was 1 in 433 patients (92.1%), 2 in 10 patients (2.1%), 3 in 24 patients (5.1%), 4 in 3 patients (0.6%).

#### sGFAP in Patients with AIS

**Fig. 1:**
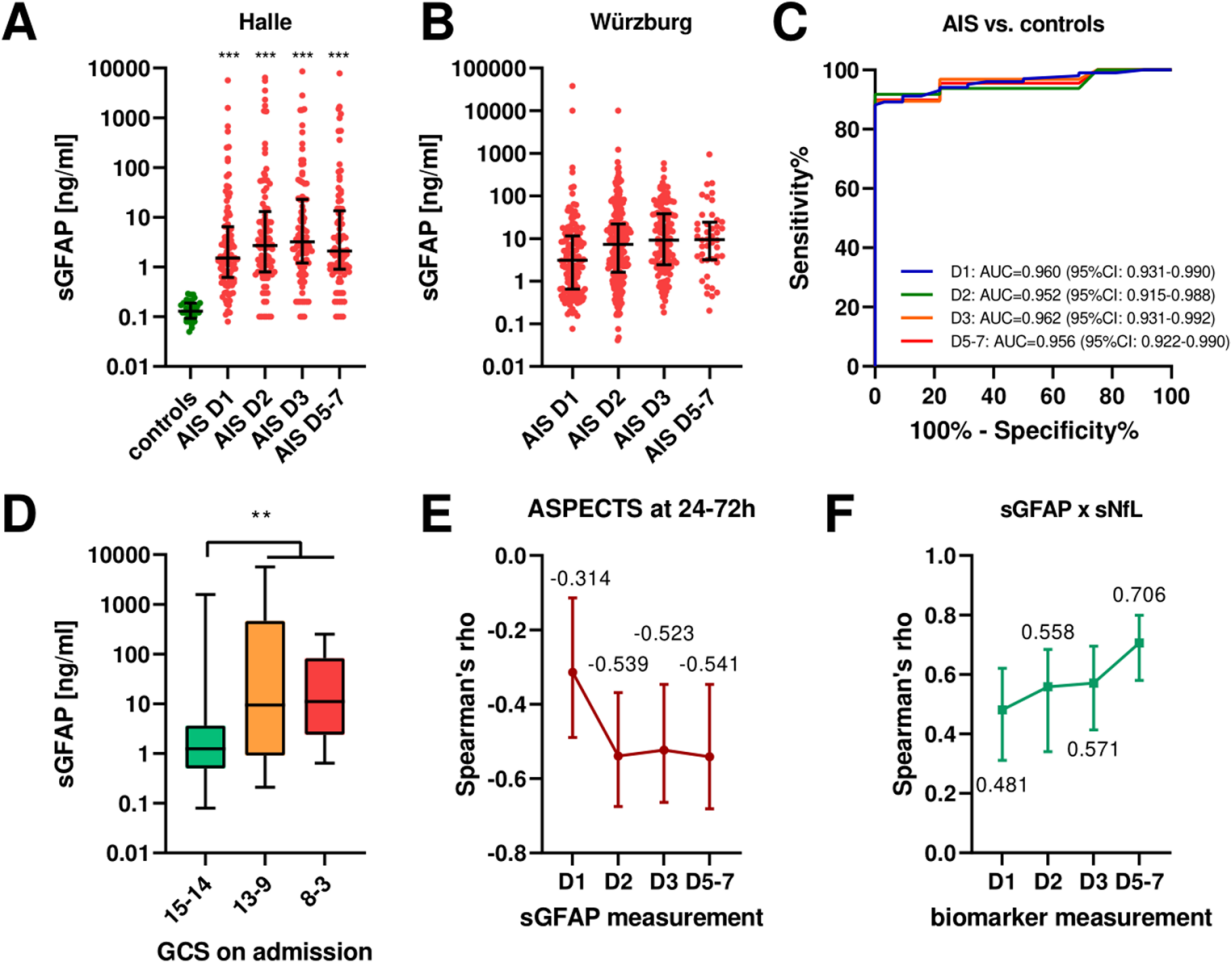
sGFAP in AIS. Biomarker levels measured at different timepoints after AIS onset in patients (**A**) from the Halle cohort (AIS *n* = 102, controls *n* = 32) and (**B**) from the Würzburg cohort (IS *n* = 470). (**C**) ROC analysis for the discrimination between AIS patients and controls. (**D**) sGFAP levels in patients with altered mental status on admission on the GCS. (**E**) Strength of the correlations between sGFAP measured at several time points and ASPECTS points on native CT after 24–72 h from AIS onset. (**F**) Strength of the correlations between sGFAP and sNfL measured at different timepoints after AIS onset. In plots A and B, lines indicate median value and interquartile range. In plots E and F, bars indicate 95% confidence intervals. *AIS* acute ischemic stroke, *ASPECTS* alberta stroke program early CT Score, *AUC* area under the curve, *GCS* glasgow coma scale, *ROC* receiver operating characteristic analysis, *sGFAP* serum glial fibrillary acidic protein, *sNfL* serum neurofilament light chain

sGFAP level at all time points was significantly higher in patients with AIS compared to controls (*p* < 0.001), and statistical significance was maintained after adjustment for age and eGFR (D1: *p* = 0.002; D2: *p* = 0.010, D3: *p* = 0.016; D5-7: *p* = 0.015) (Fig. 1A). By analysing repeated sGFAP measurements in patients from Halle, we found that sGFAP concentrations significantly increased from D1 to D2 (FDR *p* < 0.001) and to D3 (FDR *p* < 0.001) and significantly decreased from D3 to D5-7 (FDR *p* = 0.001) (Fig. 1A-B). In comparison, sNfL concentrations were steadily increased from D1 to D5-7 in both cohorts, and concentrations were significantly higher in later measurements (Supplemental Figure S2). In a subanalysis of patients undergoing blood sampling within 12 h from AIS onset [*n* = 24, median NIHSS: 16 points (IQR: 10–18 points)], we found significantly increased sGFAP and sNfL levels compared to control subjects (median sGFAP values: 1.2 ng/ml in AIS vs. 0.13 ng/ml in controls, *p* < 0.001; median sNfL values: 77.0 pg/ml in AIS vs. 7.0 pg/ml in controls, *p* < 0.001) (Supplemental Figure S2). Accuracy of sGFAP and sNfL for distinguishing AIS from control subjects was very high at all time points (including sampling < 12 h from AIS onset) with AUC values > 0.92 (Supplemental Table S2, Fig. 1C).

#### sGFAP and AIS Characteristics

Higher sGFAP concentrations correlated with clinical and radiological scores of stroke severity. In detail, sGFAP level was positively correlated with the NIHSS score at hospital admission, at 24 h, 48 h, 72 h and at discharge in both cohorts (Spearman’s rho: 0.38–0.71) (Supplemental Tables S2-S3). sGFAP level at D1 was higher in patients with altered mental status on the Glasgow coma scale (GCS < 14) at hospital admission (*p* = 0.001, Fig. 1D). We also found significant correlations between higher sGFAP and lower ASPECTS evaluated at admission and after 24–72 h (Fig. 1E, Supplemental Tables S2-S3). Admission sGFAP level was neither correlated with renal function (eGFR) in AIS patients, nor with age or BMI at any timepoint. We did not find significant correlations between sGFAP concentrations and most routine lipid metabolism, coagulation and cardiac markers (Supplemental Results, Supplemental Table S2). sGFAP and sNfL were well correlated with each other especially when measured after D2 (rho: 0.31–0.70), also accounting for renal function (Fig. 1F, Supplemental Table S1).

#### sGFAP in AIS Patients with Large Vessel Occlusion

Large vessel occlusion (LVO) was detected on admission CT angiography in 53/102 AIS patients from Halle (52.0%) and 416/470 patients from Würzburg (88.5%). sGFAP concentrations at D0 (i.e., before neuroimaging with CT-angiography) were significantly increased in AIS patients who were detected to have LVO (*n* = 17) at CT-angiography (*p* = 0.031), with an AUC for identification of LVO of 0.702 (95%CI: 0.539–0.865). Moreover, sGFAP level was increased at all timepoints in AIS patients with LVO compared to other patients (Supplemental Figure S3). Instead, sNfL level was not significantly different in AIS patients with vs. without LVO at early timepoints (D0 *p* = 0.312, D1 *p* = 0.387, D2 *p* = 0.082) and was increased in the former compared to the latter group at first at D3 and D5-7 (*p* = 0.034 for both) (Supplemental Figure S3). In the Würzburg cohort, sGFAP concentrations in first available samples were higher in patients with LVO than in other patients (*p* = 0.007, p adjusted for age, eGFR, NIHSS at admission and time-to-sampling *p* = 0.025), with an AUC value of 0.614 (95%CI: 0.539–0.690). Revascularization after MT did not impact significantly on sGFAP level changes over time in LVO-AIS (**Supplement**).

#### sGFAP and Hemorrhagic Transformation of AIS

**Fig. 2:**
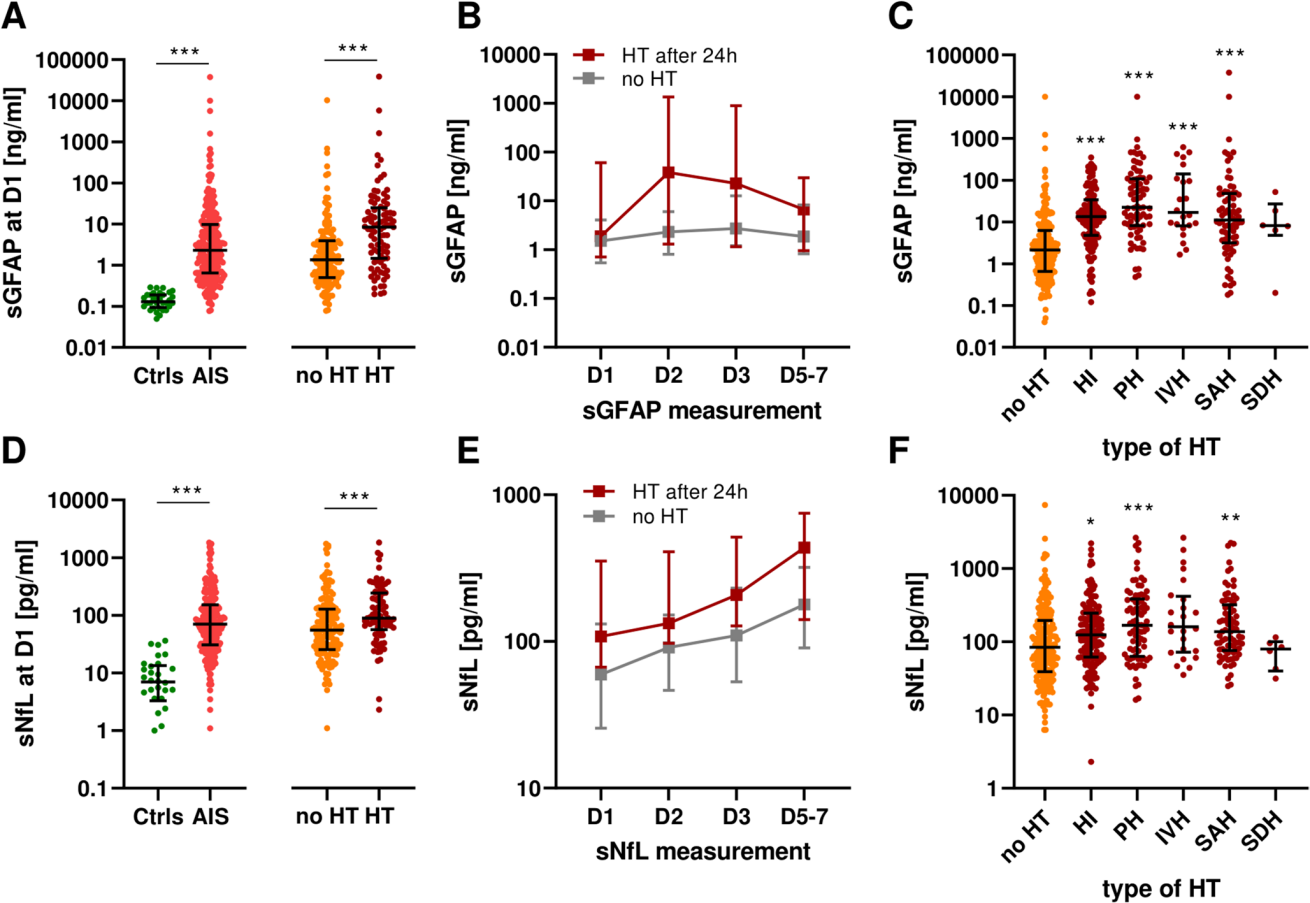
sGFAP and sNfL in patients with AIS and HT. Panels on the left illustrate early quantification (< 24 h from AIS onset, D1) of (**A**) sGFAP and **D**) sNfL in patients with AIS vs. controls and in AIS patients who developed HT vs. those who did not develop HT during hospitalization. Panels in the middle represent the temporal changes of (**B**) sGFAP and **E**) sNfL in AIS patients with early HT (i.e. at neuroimaging after 24 h from AIS onset) compared to other AIS patients. Panels on the right refer to (**C**) sGFAP and **F**) sNfL in patients with HT of different HBC types, namely hemorrhagic infarction (*n* = 144), parenchymal hematoma (*n* = 77), intraventricular hemorrhage (*n* = 22), subarachnoid hemorrhage (*n* = 75), subdural hematoma (*n* = 6). Reported p-values refer to the comparison with AIS patients without HT (*n* = 215). Lines indicate median value and interquartile range. **p < 0.01. ***p < 0.001. *AIS* acute ischemic stroke, *Ctrls* control subjects, *IVH* intraventricular hemorrhage, *HI* hemorrhagic infarction, *HT* hemorrhagic transformation, *PH* parenchymal hematoma, *SAH* subaracnoid hemorrhage, *SDH* subdural hematoma, *sGFAP* serum glial fibrillary acidic protein, *sNfL* serum neurofilament light chain

Data on HT were available for 464/470 AIS patients of the Würzburg cohort (98.7%), of which 249/464 developed HT (53.7%), and in all patients of the Halle cohort, where HT occurred in 26 of 102 patients (25.5%). Type of HT according to the Heidelberg Bleeding Classification (HBC, Würzburg cohort) [13] and timepoint of HT occurrence after AIS (Halle cohort) are reported in **Supplemental Results** and Supplemental Table S4. Early sGFAP concentrations (i.e., D1) were significantly higher in patients who developed HT during hospitalization than in other patients (*p* < 0.001) (Fig. 2A). In patients with data on HT timing, sGFAP rapidly increased from D1 to D2 and decreased lately in patients with HT after 24 h from AIS onset (*p* = 0.029) (Fig. 2B). When analyzing HBC types of HT, sGFAP levels were increased in patients who developed any HT except for subdural hemorrhage than in AIS patients who did not develop HT (*p* < 0.001) (Fig. 2C). As a comparison, early sNfL levels (i.e., D1) were increased in AIS patients who developed HT too, but biomarker increased after HT occurred was more pronounced from D3 onwards (*p* < 0.01) (Fig. 2D-E). Moreover, according to the HBC subtype of HT, sNfL was more elevated in case of hemorrhagic infarction (*p* = 0.038), parenchymal hematoma (*p* < 0.001) and subdural hematoma (*p* = 0.002) (Fig. 2F).

#### sGFAP and Infections in AIS Patients

In the Halle cohort, 33 patients (32.4%) had an infection during the sampling period, most frequently pneumonia (detailed data on type and timing in **Supplement**). Given the above-mentioned relationship between HT and higher biomarker values, we performed analysis on infection status in AIS patients who did not develop HT to avoid misinterpretation of results. sGFAP and sNfL were significantly correlated with laboratory parameters of inflammation (i.e., C reactive protein and leucocyte count) measured at the same time points of biomarker quantification, especially after infection onset (Supplemental Table S4). sGFAP level at all timepoints was significantly higher in AIS patients with infections than in other patients (D1 *p* = 0.003, *p* < 0.001 for other timepoints), while sNfL was increased from D2 samples onwards (**Supplement**, Supplemental Figure S4).

#### sGFAP and Functional Outcome at 3 Months

**Fig. 3:**
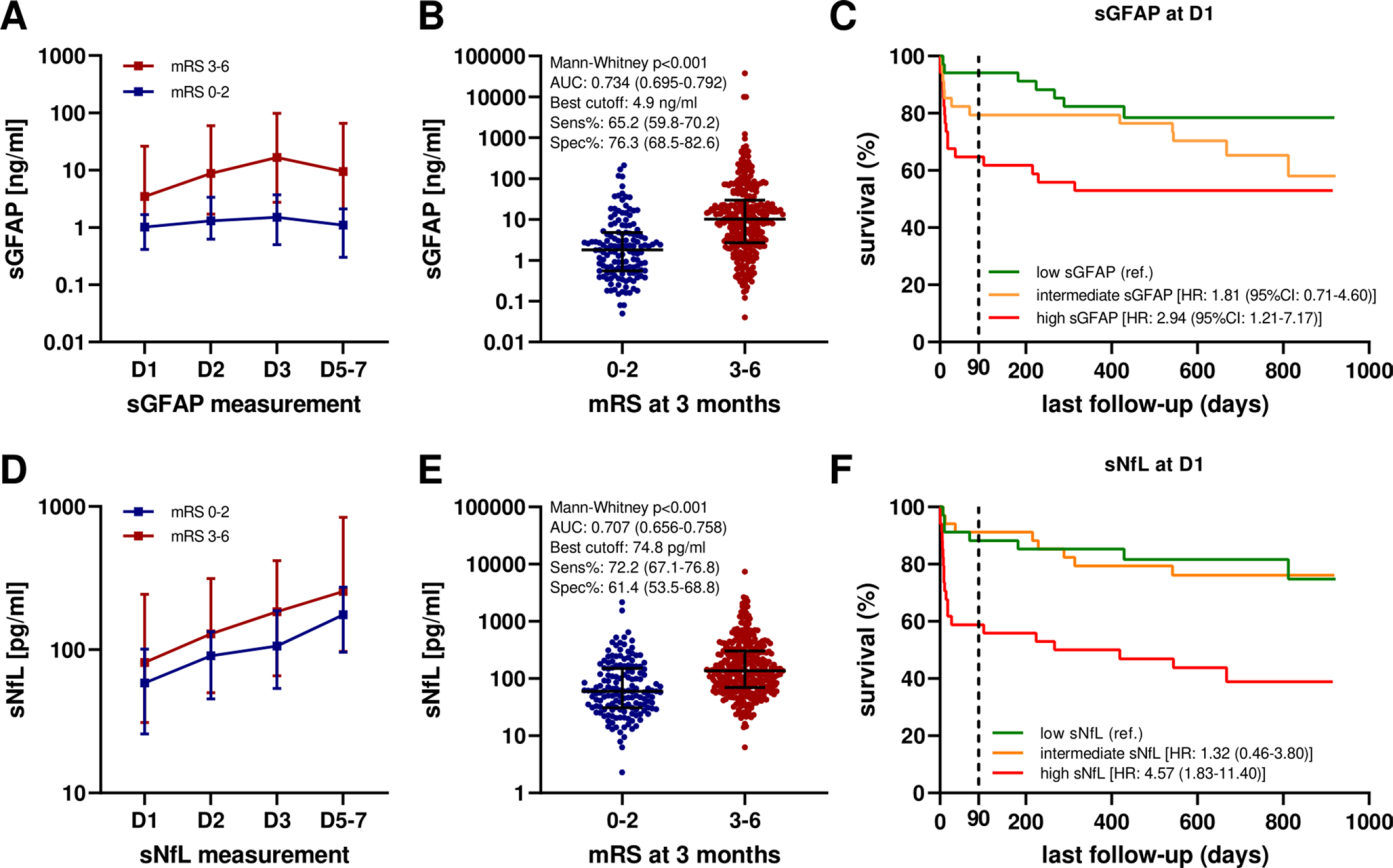
sGFAP and clinical outcome after AIS. Panels illustrate the temporal changes of (**A**) sGFAP and **D**) sNfL over the first week after onset in AIS patients of the Halle cohort with mRS at 3 months of 3–6 vs. 0–2 (or unchanged to pre-stroke mRS). Panels in the middle represent first available concentrations of (**B**) sGFAP and **E**) sNfL in patients of the Würzburg cohort. Data reported refer to ROC analysis for the discrimination between the two mRS groups (3–6 vs. 0–2 or unchanged to pre-stroke mRS) and best cutoff with corresponding sensitivity (Sens%) and specificity (Spec%) were calculated by maximizing the Youden index. Lines indicate median value and interquartile range. Panels on the right refer to Kaplan-Meier analysis for (**C**) sGFAP and F) sNfL in patients of the Halle cohort [median follow-up: 576 days (IQR: 239–807, range: 1–922 days)]. HR values derived from univariable Cox regression analysis (results of univariable and multivariable Cox analysis in Supplemental Table S8). Results were calculated on last follow-up, we also added a dashed line at 90 days of follow-up for better interpretation of data. *AIS* acute ischemic stroke, *HR* hazard ratio, *mRS* modified rankin scale,*ROC* receiver operating characteristic, *sGFAP* serum glial fibrillary acidic protein, *sNfL* serum neurofilament light chain

In the Halle cohort, all 102 AIS patients had follow-up data at 3 months, of which 51 had mRS > 2 (50.0%). sGFAP level at baseline was increased in AIS patients with mRS 3–6 vs. 0–2 or unchanged to pre-stroke mRS at all individual timepoints (*p* < 0.001) (Table 2; Fig. 3A), but statistical significance was lost at multivariable analysis (i.e. age, eGFR and NIHSS on admission) (complete data in Supplemental Table S6). Results were similar after excluding AIS patients who developed HT or those with infection, i.e., significantly higher biomarker level in those with poor vs. those with good outcome only at unadjusted analysis. Similar results were observed for sNfL, with marginal significance at D3 (*p* = 0.048) and D5-7 (*p* = 0.499) after adjustment for covariables (Table 2; Fig. 3, Supplemental Table S7). Results were not different after excluding patients who developed HT or according to infection status, especially for D3 (*p* = 0.048) and D5-7 samples (*p* = 0.025).

**Table 2:** Serum biomarker levels in IS patients according to mRS at 3 months

sGFAP [ng/ml]	Halle (*n* = 102)			Würzburg (*n* = 470)		
	mRS 0–2 or unchanged to pre-stroke mRS (*n* = 51)	mRS 3–6 (*n* = 51)	*p*	mRS 0–2 or unchanged to pre-stroke mRS (*n* = 246)	mRS 3–6 (*n* = 324)	*p*
D1	1.1 (0.5–1.7)	3.5 (1.0–23.8)	0.001	0.7 (0.4–2.6)	5.8 (1.6–15.2)	< 0.001
D2	1.3 (0.7–3.4)	8.8 (1.9–55.7)	< 0.001	2.2 (0.8–5.6)	13.1 (3.0–42.6)	< 0.001
D3	1.7 (0.6–3.6)	16.6 (2.8–79.0)	< 0.001	3.1 (1.5–14.0)	16.3 (3.5–44.6)	0.002
D5-7	1.1 (0.5–2.1)	9.5 (2.1–52.1)	< 0.001	2.3 (0.6–14.5)	11.4 (4.0–26.6)	0.028
sNfL [pg/ml]						
D1	46.2 (22.6–98.9)	85.8 (53.7–329.0)	0.003	32.9 (20.7–64.5)	90.7 (54.7–200.5)	< 0.001
D2	73.4 (34.8–129.0)	140.5 (75.9–346.3)	< 0.001	66.9 (35.4–146.0)	124.0 (63.9–253.0)	< 0.001
D3	87.0 (45.1–178.8)	221.0 (91.7–462.0)	< 0.001	81.0 (51.6–199.0)	188.0 (115.5–352.0)	< 0.001
D5-7	141.5 (86.4–232.5)	322.0 (148.0–881.5)	< 0.001	275.5 (252.8–325.5)	610.0 (336.0–1419.8)	0.020

Reported p-values derived from Mann-Whitney *U* test

*mRS* modified rankin scale, *sGFAP* serum glial fibrillary acidic protein, *sNfL* serum neurofilament light chain protein

In the Würzburg cohort, 319 patients out of 460 with available follow-up data at 3 months had poor functional outcome (mRS > 2, 69.3%). Patients with good or poor functional outcome at follow-up (*p* = 0.284) and survivors vs. non-survivors (*p* = 0.677) did not significantly differ in the time (in days) from AIS onset to first blood sampling. In the first available measurement, higher sGFAP and sNfL concentrations were significantly associated with poorer clinical outcome (i.e., mRS > 2 at 3 months) also at multivariable analysis, with moderate discriminative accuracy at ROC analysis [area under the curve for mRS 3–6 vs. 0–2: 0.734 (95%CI: 0.695–0.792) for sGFAP and 0.707 (95%CI: 0.656–758) for sNfL] (Fig. 3B and D; Table 2). Significant associations at multivariable analysis were found for distinct time points of biomarker quantification (Supplement, Supplemental Table S6-S7).

#### Survival Analysis

For survival analysis in the Halle cohort, AIS patients were followed up for a median time of 576 days (IQR: 239–807, range: 1–922 days). Seventeen (16.7%), 22 (21.6%) and 35 (34.3%) patients died during hospitalization [median time from onset to death: 8 days (IQR: 6–10 days)], at 3 months and at the end of follow-up, respectively. In the Würzburg cohort, 117/470 patients died during hospitalization (24.9%) and 156/460 patients died before 3 months (33.9%, *n* = 10 with missing data on 3-month survival). AIS non-survivors of both cohorts (i.e. both intra-hospital and 3-month mortality) had increased levels of sGFAP and sNfL already at D1 than AIS survivors (*p* < 0.01 for all timepoints) in both cohorts. Statistical significance survived after excluding patients who developed HT. More elevated sGFAP concentration in first available samples was associated overall mortality during hospitalization (*p* = 0.004) and at 3 months (*p* = 0.004) at adjusted analysis (i.e., for age, eGFR, time-to-sampling and NIHSS on admission). By analysing single timepoints of quantification, sGFAP at D3 was associated with intra-hospital (*p* = 0.011) and 3-month mortality (*p* = 0.025) in multivariable models (Supplemental Table S6). As a comparison, sNfL was associated with AIS mortality as single-quantification and at multiple timepoints (especially D1 and D3, Supplemental Table S7). At Kaplan-Meier analysis, AIS patients with higher levels of sGFAP and sNfL (i.e.3rd tertile) at any timepoint were significantly associated with shorter survival times compared to patients with lower biomarker levels (i.e. 1 st tertile), also after considering for covariables including HT (hazard ratio, HR: 2.94–5.91 for 3rd vs. 1 st sGFAP tertile, HR: 4.57–7.73 for 3rd vs. 1 st sNfL tertile) (Fig. 3C and F; Table 3).

**Table 3:** Results of Cox regression analysis with survival data

sGFAP	2nd tertile vs. 1 st tertile		3rd tertile vs. 1 st tertile	
	HR (95%CI)	*p*-value	HR (95%CI)	*p*-value
D1	1.81 (0.71–4.60)	0.214	2.94 (1.21–7.17)	0.018
D2	0.93 (0.34–2.57)	0.893	2.80 (1.20–6.55)	0.018
D3	1.69 (0.55–5.16)	0.360	5.18 (1.90–14.14)	0.001^a, b^
D5-7	2.24 (0.69–7.27)	0.180	5.91 (1.97–17.73)	0.001^a, b^
sNfL				
D1	1.32 (0.46–3.80)	0.609	4.57 (1.83–11.40)	0.001
D2	2.05 (0.62–6.80)	0.242	6.82 (2.31–20.08)	< 0.001^a, b^
D3	2.02 (0.61–6.71)	0.252	6.52 (2.20–19.30)	< 0.001^a, b^
D5-7	3.33 (0.91–12.09)	0.068	7.73 (2.25–26.59)	0.001^a, b^

Cox regression was computed with biomarker tertile groups by taking the lowest tertile as reference. Together with p-values from univariate analysis, statistical significance of multivariable models are reported as follows:

a) statistically significant after adjustment for age, eGFR, NIHSS on hospital admission

b) statistically significant after adjustment for age, eGFR, NIHSS on hospital admission and HT

*eGFR* estimated glomerular filtration rate, *HR* hazard ratio, *HT* hemorrhagic transformation, *NIHSS *national institute of health stroke scale, *sGFAP* serum glial fibrillary acidic protein, *sNfL* serum neurofilament light chain protein

#### sGFAP in AIS Due to BA Occlusion

Given the lack of data on sGFAP and sNfL in patients with AIS due to basilar artery (BA) occlusion, we conducted an exploratory analysis in this subgroup (Würzburg *n* = 33; Halle *n* = 5, not analysed due to small sample size). sGFAP level was similar in patients with BA occlusion than in patients with occlusion of other cerebral arteries, did not significantly differ in samples drawn at different timepoints, were correlated at D2 with admission NIHSS score (rho = 0.738, *p* = 0.002) and were higher in patients who died during hospitalization than in other patients (*p* = 0.004) (further details and data on sNfL in **Supplemental Results** and Supplemental Figure S5).

## Discussion

In this study, we aimed to investigate the role of sGFAP as an astrocytic biomarker in AIS and its predictive value in two independent German prospective AIS cohorts. Our data show that sGFAP level is elevated within the first 24 h from AIS onset, progressively increases in the first days after AIS with peak concentrations at D3 and remained stable or decreased at D5-7. Higher biomarker concentrations were correlated with greater disease severity on clinical and radiological scores (NIHSS and ASPECTS, respectively), with higher risk of HT, systemic infection and intra-hospital mortality after AIS, as well as with poorer clinical outcome at 3 months. These results deepen our understanding of astroglial markers in acute and post-acute phases of AIS and suggest sGFAP as an informative marker for early risk stratification of AIS patients.

sGFAP concentrations were remarkably elevated in AIS patients compared to control subjects. Although other authors reported only slight differences between AIS and AIS mimics by measuring GFAP with electrochemiluminescence-based immunoassay [16], our findings are in line with previous results obtained on the Simoa platform [2–4]. The fact that sGFAP was remarkably increased since the first hours after onset especially in severe AIS (e.g., NIHSS > 10 points) raises questions on its use in real-world contexts for the discrimination between hemorrhagic and ischemic stroke [9]. Moreover, we observed a remarkable elevation of sGFAP concentrations also after excluding patients who developed HT. This suggests that sGFAP elevation most likely reflects AIS-related mechanisms, for example ischemic astrocyte injury, blood-brain barrier dysfunction and upregulated astroglial activity near ischemic regions [17]. In comparison to sNfL, which increases later during the disease course (i.e., after days after AIS onset), sGFAP may represent an early marker of vascular brain injury, similarly to what observed after acute brain injury of other nature, such as traumatic brain injury (TBI) [18, 19] and neurosurgery for glioblastoma [20]. However, with respect to head trauma where peak GFAP concentrations are reached within 24 h from onset [18, 19], sGFAP increased significantly up to about 72 h after onset. As another difference, the magnitude of sGFAP elevation in AIS seems to be greater than in TBI (10- up to 30-fold vs. 5- up to 15-fold, respectively), thus possibly justifying the delayed peak. Alternatively, AIS-specific mechanisms could contribute to the sGFAP elevation after 24 h, but these hypotheses need experimental validation.

We observed that sGFAP level is highly sensitive and temporally related to HT occurrence of almost all HBC subtypes and, for intraparenchymal HT, sGFAP was progressively higher in patients with larger hemorrhages (e.g. HBC type 1c or 2) compared to small petechiae (i.e. HBC 1a). In comparison, sNfL response to HT was seen later, hence showing preliminarily less clinical utility for early detection of HT after AIS and possibly reflecting secondary neurodegeneration following HT. sGFAP may thus be a promising tool for monitoring neuroinflammatory processes after AIS also considering the dozens of clinical trials evaluating immunomodulatory drugs in AIS [21]. Also, this turns particularly relevant for increasing clinical confidence in timing the start or restart of antithrombotic therapy after AIS [10, 22], but biomarker-guided strategies for establishing antithrombotic regimens after AIS need experimental validation in targeted trial settings.

On another level, we provided preliminary data on sGFAP in blood samples collected before imaging and acute therapy, and found that sGFAP was significantly more elevated in AIS patients with LVO compared to patients with other types of AIS. If used alone, clinical scores for identifying patients with AIS due to LVO in pre-hospital settings have a rather high misclassification rate [23]. To ameliorate pre-hospital triaging of patients and to indicate transfer to MT-capable centers, future studies should evaluate the combination of sGFAP, as well as sNfL and other candidate markers, with indicative clinical score for suspecting AIS due to LVO before neuroimaging [24, 25]. In our analysis, early and repeated sGFAP measurement turned out to carry prognostic value for short- and middle-term outcomes. Indeed, sGFAP level remained stable over time in the lower concentration range in patients with rapid clinical improvement after stroke therapy and no intra-hospital complications (median values < 3 ng/ml), whereas there were sharply increased values (median values up to 10–15 ng/ml) within few days in patients who had HT or died during hospitalization. Moreover, sGFAP elevation was also temporally related to occurrence of systemic infections such as pneumonia. The interpretation of these results remains to date mainly speculative given the overall small sample size and the lack of preclinical data. More elevated sGFAP concentrations most likely reflect more severe vascular brain injury which may then predispose to systemic infections. Alternatively, future studies should evaluate whether sGFAP could reflect also infection-driven astroglial response in the central nervous system, hence providing a possible explanation for the association between pneumonia and poorer outcomes after ischemic stroke. We observed that patients with poorer clinical outcome at follow-up showed higher sGFAP values during the first week after AIS. Intra-hospital complications impact heavily on clinical outcomes after IS, but sGFAP still retained prognostic value in patients without HT or infections. For predicting the risk of functional disability and/or all-cause mortality at 3 months, sNfL showed greater robustness at several time points of biomarker quantification and possibly a higher overall value, especially when measured after few days from onset. Here, results from large multinational cohorts may help the implementation of sNfL for better prognosticating AIS [26], and similar efforts will be needed for sGFAP too. Also, data on sGFAP in outpatients for assessing risk of stroke recurrence, for monitoring antithrombotic therapies as well as in primary AIS prevention are still lacking, and real-world studies will need to evaluate the net clinical benefit of implementing fluid biomarkers for rapid stroke prognostication [27], either alone or in combination with already established clinical scores [28]. One of the strengths of our work relies on the multicentric design and cohorts with different AIS characteristics and inclusion criteria, thus being representative of the real-world AIS population. Most results could be replicated in both cohorts, even though patients recruited in Würzburg had more severe AIS at hospital admission (NIHSS score of 13 points vs. 7 points of patients recruited in Halle) and had more frequently AIS due to LVO (88.5% vs. 52.0%). Moreover, in a preliminary analysis, we investigated biomarker levels in AIS due to BA occlusion, which is frequently underrepresented in biomarker and clinical studies. In this subpopulation, we found similar results as in patients with AIS due to middle cerebral artery occlusion, namely correlations between sGFAP level and NIHSS score as well as higher concentrations in patients who died during hospitalization. These preliminary results should encourage further studies to better assess the robustness of prognostic biomarkers in different AIS subtypes. Further, we provided detailed data on onset and type of HT and systemic infections, thus letting us explore the temporal association between biomarker changes and neurological but also systemic complications after AIS. As limitations of our study, we acknowledge that our control group does not picture the neurological diseases that represent typical differential diagnoses of AIS in which elevated biomarker values can be found, such as in epileptic seizures [29]. Hence, our findings tend to overestimate the discriminative accuracy of such biomarkers and cannot be taken alone for inferences on their diagnostic value for AIS, which was out of the scope of this present study. Notwithstanding, biomarker concentrations in the control subjects of our cohort were in the same range of previous cohorts without neurological diseases measured with the same assays [30]. Moreover, given that sGFAP concentrations increase approximately 2% per year in the healthy population [15], the magnitude of sGFAP elevation observed in AIS (more than 10-fold increase) is most likely attributable to AIS pathophysiology rather than to the age gap between our disease and control groups. Similarly, the differences in the inclusion criteria and blood sampling protocols could have contributed to the variability of some results, for example on the predictive value of the biomarker for 3-month outcomes. Here, analysis in the Würzburg cohort were overall more robust after adjustment for covariables, which could be due to both a larger sample size and a greater AIS severity of patients included. Second, we lacked detailed neuroimaging data other than the ASPECTS, which does not fully capture the heterogeneity of infarct burden at the individual level. We found negative correlations between sGFAP levels and ASPECTS points, i.e., higher biomarker concentrations were correlated with larger expected infarct volumes. Correlations were found already from very early marker quantifications (within 24 h from onset) and became stronger in the following days. Even if the analysis was limited by the lack of data on final infarct volume size at CT or magnetic resonance imaging (MRI, see also below the study limitations), the ASPECTS represents a well-established and clinically meaningful surrogate marker of infarct extent. Each ASPECTS value is inherently tied to different infarct volumes and shows correlations with functional outcomes [31] and final infarct volume [31, 32]. Still, this relationship may not universally apply to smaller infarct volumes, is not strictly linear and may be influenced by factors such as lesion location, timing of imaging, and imaging modality. Despite the independent contribution of sGFAP and sNfL for prognosticating AIS [7], the association between fluid biomarkers and core/penumbra volumes or white matter changes [33], as well as novel imaging strategies for central nervous system inflammation [21], remain unexplored. Further, given the age of the included patients (mean: 70.9 and 74.6 years in the two cohorts), information on the pre-existing vascular brain injury would have been helpful for better data interpretation. Here, future studies should include MRI measures, or CT-based estimates [34], of small vessel disease to assess the impact of microangiopathic injury burden on biomarker dynamics over time. This would aid understanding whether and at which extent fluid biomarkers hold predictive value for clinical outcomes beyond neuroimaging. Third, inclusion criteria of both cohorts were centered on AIS, hence blood samples for direct comparisons with ICH were not available. Moreover, blood samples collected provided a rather granular view of biomarker kinetics during post-acute phases. More frequent blood sampling during the first days, ideally every hour or few hours, would help defining in detail the temporal course of marker concentrations and the best timepoint of quantification for clinical purposes. This may have impacted also on interpretation of results derived from survival analysis. In fact, early in-hospital deaths may have biased the longitudinal analysis given that no complete sample sets during the observation period was available. Fourth, despite the detailed data on HBC type of HT, we could not directly analyze the associations between biomarker trajectories and HT onset for each HBC subtype, which would be of great clinical utility.

In conclusion, we found that sGFAP concentrations are elevated in patients with AIS, progressively increase over time and are associated with clinical and radiological disease severity. Higher sGFAP level after AIS may inform on the risk of intra-hospital complications, such as HT and infections, as well as on the likelihood of functional disability and mortality at follow-up evaluation.

## Supplementary Information

Below is the link to the electronic supplementary material.

Supplementary Material 1 (DOCX 44.4 KB)

## Data Availability

Anonymized data will be shared with qualified investigators on reasonable request to the corresponding author.
