# Supplementary material for: Temporal Pattern and Clinical Value of Serum GFAP in Acute Ischemic Stroke: Results from Two Prospective German Cohorts
Source: Transl Stroke Res. 2026 Jun 15;17(3):67. doi: 10.1007/s12975-026-01458-1 (PMC13269383; doi:10.1007/s12975-026-01458-1)
Supplement: Supplementary file 1 — Supplementary Material 1 (DOCX 44.4 KB) [file 12975_2026_1458_MOESM1_ESM.docx]

**SUPPLEMENTAL METHODS**

**Collected data of the study cohorts.** In all patients, we included baseline demographic, clinical, radiological and biochemical data, including disease severity (i.e., NIHSS score), comorbidities, the Alberta Stroke Program CT Score (ASPECTS), laboratory parameters of renal function [as estimated glomerular filtration rate (eGFR), as calculated with the Chronic Kidney Disease – Epidemiology Collaboration (CKD-EPI), formula (<https://www.kidney.org/ckd-epi-creatinine-equation-2021>)] and systemic inflammation (i.e., C reactive protein, CRP, leucocyte count). Moreover, we collected additional data during the hospitalization period, such as serial evaluation of NIHSS score (after 24, 48, 72 hours and at discharge) and ASPECTS (after 24 hours), as well as occurrence of hemorrhagic transformation of AIS (HT) and systemic infections (further details below). Concerning AIS characteristics, data collected included IS etiology according to the Trial of Org 10172 in Acute Stroke Treatment (TOAST) classification, namely large-artery atherosclerosis (LAA), cardioembolism (CE), small-vessel disease (SVD), other determined etiology and cryptogenic AIS. Further, we distinguished patients with different AIS territory according to the infarct localization, namely anterior cerebral artery (ACA), middle cerebral artery (MCA), posterior cerebral artery (PCA), vertebral artery (VA) and basilary artery (BA). Data on acute stroke therapy included the number of patients undergoing intravenous thrombolysis (IVT) and/or mechanical thrombectomy (MT), as well as recanalization success as modified Thrombolysis In Cerebral Ischemia (mTICI) score. At 90-day follow-up, data on modified Rankin scale (mRS) and all-cause mortality were collected through clinical visit or, if not possible, telephone interview.

**Neuroimaging data acquisition and analysis.** As a surrogate marker of the infarcted tissue volume, we collected data on the ASPECTS on native non-contrast CT upon hospital admission (Halle cohort n=95/102, 93.1%; Würzburg cohort n=427/470, 90.9%) and after 24 hours (Halle cohort n=94/102, 92.2%). Data on final infarcted lesion size on CT or magnetic resonance imaging (MRI) were not available for this study. In patient undergoing MT, we collected data on reperfusion status according to the modified Treatment In Cerebral Infarction (mTICI) score, where mTICI scores of 2b, 2c and 3 were considered as successful recanalization. Also, we had available data on hemorrhagic transformation (HT) of IS. In the Halle cohort, we distinguished patients who developed intracerebral hemorrhage (ICH) and subarachnoid hemorrhage (SAH) at follow-up imaging during hospitalization (i.e. CT and/or MRI) and classified HT into asymptomatic and symptomatic if associated with a clinical worsening of at least 4 NIHSS points or death. Moreover, data on the time between AIS onset and HT detection on neuroimaging was also collected. In the Würzburg cohort, HT was classified according to the Heidelberg Bleeding Classification (HBC) subtypes (n=464/470, 98.7%), whereas data on HT or on symptomatic/asymptomatic HT was not available.

**SUPPLEMENTAL RESULTS**

**Associations between sGFAP and demographic and laboratory variables.**

Halle cohort. sGFAP concentrations were not significantly correlated with age or body-mass index (BMI) in AIS patients (p>0.05 at all timepoints). They were negatively correlated with renal function (eGFR) at D3 (rho=-0.229, p=0.026) and D5-7 (rho=-0.266, p=0.013). Instead, they were not correlated with BMI, triglycerides, cholesterol (total cholesterol, high-density lipoproteins, low-density lipoproteins), coagulation parameters (international normalized ratio, INR), troponin T, N-terminal prohormone of brain natriuretic peptide (NT-pro-BNP) (**Supplemental Table S2**). We found moderate to strong correlations between sGFAP at different timepoints of quantification with each other (rho values: 0.68-0.90) as well as significant negative correlations between sGFAP and hemoglobin levels (rho values: -0.22 - -0.38) (**Supplemental Table S2**).

Würzburg cohort. We did not find significant correlations between sGFAP and age or renal function (**Supplemental Table S3**). sGFAP concentrations correlated with each other at different timepoints but with low sample size (n≤26) (**Supplemental Table S3**). Similarly, we found significant correlations between sGFAP and sNfL at different timepoints (rho: 0.41-0.80) (**Supplemental Table S3**).

**sGFAP in patients with successful recanalization.** In the Würzburg cohort, 311/359 (86.6%) patients who received MT had successful revascularization (mTICI of 2b, 2c or 3), and sGFAP levels did not significantly differ between the two groups (p=0.068). We could not assess the effects of unsuccessful revascularization on biomarker levels in the Halle cohort given that MT gave successful reperfusion in all patients except one (46/47, 97.8%).

**Hemorrhagic transformation of AIS.** In patients from Halle, HT (26/102, 25.5%) consisted of 4 cases with subarachnoid hemorrhage (SAH, 4/102, 3.9%) and 22 cases of intracerebral hemorrhage (ICH, 21.6%). SAH was asymptomatic in all cases, whereas ICH was symptomatic in 5 cases (5/102 of all IS patients, 4.9%; 5/22 of all HT cases, 22.7%). HT occurred in most cases 1 day after onset (16/25, 64.0%), including all symptomatic ICH events. Otherwise, asymptomatic HT was observed at neuroimaging after 2 (n=1), 3 (n=1), 4 (n=5), 5 (n=1), 6 (n=1) and 7 days (n=1) after stroke onset. In patients from the Würzburg cohort, the frequency of Heidelberg Bleeding Classification (HBC) HT subtypes is reported in **Supplemental Table S4**, whereas data on timepoint of HT and whether HT was symptomatic or not was not available.

**Infections in patients with AIS.** Of 33 IS patients of the Halle cohort who developed infections during the hospitalization period (32.4%), most of patients had pneumonia (n=25, 75.8%), 6 patients had urinary tract infection (18.1%), and 2 patients had infective endocarditis (which were identified as the cause of IS, 6.1%). In their medical history, AIS patients of the Halle cohort did not have relevant hematological disorders, except for one patient with a history of multiple myeloma. This patient did not develop infections during hospitalization for AIS but, to avoid misinterpretation of results, was excluded from analysis of inflammation parameters. Moreover, to avoid misinterpretation due to HT-related increase of sGFAP, we analyzed biomarker levels in AIS patients with infections compared to other patients after excluding patients who developed HT. This exploratory analysis of AIS patients without HT included 58 patients with no infection, 11 cases with pneumonia (n=1 occurred at D1, n=5 at D2, n=3 at D3), 4 cases of urinary tract infection (n=3 occurred at D3, n=1 at D4) and 2 cases of infective endocarditis. sGFAP was increased at all timepoints in AIS patients with infections compared to other patients (D1 p=0.003, p<0.001 for other timepoints), and association between sGFAP and infection status was still significant at D5-7 independently from admission NIHSS score (p=0.025) or the presence of LVO (p=0.008) in multivariable analysis (i.e., adjusted for age, eGFR and NIHSS score on admission or LVO). At the same analysis, significant associations were observed for sNfL at D3 (p=0.029) and D5-7 (p=0.017). As a comparison, we also illustrated temporal changes of C reactive protein (CRP) concentrations and leucocyte count (**Supplemental Figure S4**). Moreover, as an illustrative example, we visualized concentrations of all biomarkers (sGFAP, sNfL, CRP, leucocyte count) in patients who developed pneumonia at different timepoint to evaluate biomarker kinetics according to infection onset (**Supplemental Figure S4**). We did not perform quantitative analysis between subgroups given the very small sample size of subgroups (n<6).

**sGFAP in patients with AIS due to BA occlusion.** Patients with BA occlusion (BAO) had blood samples drawn at D1 in 10 cases, at D2 in 15 cases and at D3 in 9 cases (one patient had 2 blood samples drawn at D1 and D3, respectively). Patients with BAO had sGFAP concentrations that were not significantly different to AIS patients with vessel occlusion in other supply territories including the reference group (MCA), and sGFAP concentrations in BAO patients were similar at D1, D2 and D3 (**Supplemental Figure S5**). Correlations with radiological metrics of AIS severity were not possible given the lack of data on ASPECTS of the posterior circulation. Even if underpowered due to small sample size, correlation analysis with NIHSS score revealed that the NIHSS score on admission was significantly correlated with sGFAP measured after at least 24 hours from onset (i.e. D2, n=15, rho=0.738, p=0.002). Data on 3-month follow-up was available in 31 patients with BAO, of which 9 had good functional outcome (mRS of 0-2 or unchanged to pre-stroke mRS, 29.0%) and 22 had poor outcome (mRS >2, 71.0%; of which 15 did not survive, 48.4%). Patients with poor functional outcome had nominally higher but not significantly different first sGFAP concentrations than other patients (p=0.089). sGFAP level was higher in patients with BAO who died during hospitalization (p=0.004) (**Supplemental Figure S5**) but were not different when accounting 3-month mortality (p=0.202). As a comparison, we found no significant differences of sNfL levels according to stroke territory nor relevant changes over time in patients with BAO. Moreover, sNfL was not significantly correlated with NIHSS score on admission or with patients with better vs. worse outcomes (i.e., mRS 3-6 vs. 0-2, intra-hospital mortality, mortality at 3 months) (**Supplemental Figure S5**).

**SUPPLEMENTAL FIGURES**

**Supplemental Figure S1. Full study protocol and selection process of the Würzburg cohort.**

**Supplemental Figure S2.** A) sNfL in patients with AIS. Biomarker concentrations measured at different timepoints after stroke onset in patients from the Halle cohort (IS n=102, controls n=32) and from the Würzburg cohort (IS n=470). B) sGFAP and sNfL concentrations in 24 AIS patients undergoing blood sampling within 12 hours from AIS onset to demonstrate hyperacute kinetics of fluid biomarkers. AIS patients had median NIHSS values of 16 points (interquartile range: 10-18 points). Lines indicate median value and interquartile range. ***p<0.001.

**Abbreviations.** AIS: acute ischemic stroke; NIHSS: National Institute of Health Stroke Scale; sGFAP: serum glial fibrillary acidic protein; sNfL: serum neurofilament light chain.

**Supplemental Figure S3. sGFAP and sNfL in AIS patients with vs. without LVO.** Concentrations of A) sGFAP and B) sNfL in IS patients with LVO compared to other patients. In the panels on the left, biomarker concentrations refer to blood samples obtained in the emergency department before CTA. *p<0.05. ***p<0.001.

**Abbreviations.** CTA: computed tomography – angiography; LVO: large vessel occlusion; sGFAP: serum glial fibrillary acidic protein; sNfL: serum neurofilament light chain.

**Supplemental Figure S4. sGFAP, sNfL, CRP and leucocyte count in AIS patients with systemic infections.** Biomarker level in A) patients with infections vs. those without infections and B) patients who developed pneumonia at different timepoints vs. those without infections.

**Abbreviations.** AIS: ischemic stroke; CRP: C reactive protein; sGFAP: serum glial fibrillary acidic protein; sNfL: serum neurofilament light chain.

**Supplemental Figure S5. sGFAP and sNfL in patients with basilary artery occlusion.** A) Biomarker levels in patients with AIS localization in different arterial territories. 2) Temporal changes of serum biomarkers in patients with BA occlusion. C) Correlations between biomarker concentrations and NIHSS score on hospital admission. D) Biomarker level in patients who died during hospitalization vs. in those who survived until discharge. Lines indicate median value and interquartile range. **p<0.01.

**Abbreviations.** ACA: anterior cerebral artery; BA: basilary artery; IS: ischemic stroke; MCA: middle cerebral artery; NIHSS: National Institute of Health Stroke Scale; PCA: posterior cerebral artery; sGFAP: serum glial fibrillary acidic protein; sNfL: serum neurofilament light chain.

**SUPPLEMENTAL TABLES**

**Supplemental Table S1.** Diagnostic accuracy of sGFAP and sNfL for discriminating patients with AIS from control subjects in the Halle cohort.

| **sGFAP** | **AUC (95%CI)** | **best cutoff** | **sensitivity% (95%CI)** | **specificity% (95%CI)** | **p** | **adjusted p*** |
| --- | --- | --- | --- | --- | --- | --- |
| <12 h | 0.932 (0.854-1.00) | 0.285 | 79.2 (59.5-90.8) | 96.9 (84.3-99.8) | <0.001 | - |
| D1 | 0.960 (0.930-0.990) | 0.295 | 88.2 (80.6-93.1) | 100.0 (89.3-100.0) | <0.001 | 0.002 |
| D2 | 0.951 (0.914-0.988) | 0.295 | 91.8 (84.6-95.8) | 100.0 (89.3-100.0) | <0.001 | 0.010 |
| D3 | 0.961 (0.931-0.992) | 0.295 | 89.5 (81.7-94.2) | 100.0 (89.3-100.0) | <0.001 | 0.016 |
| D5-7 | 0.955 (0.921-0.990) | 0.295 | 89.9 (81.9-94.6) | 100.0 (89.3-100.0) | <0.001 | 0.015 |
| **sNfL** |  |  |  |  |  |  |
| <12 h | 0.936 (0.874-0.998) | 15.2 | 91.7 (74.2-98.5) | 83.3 (66.4-92.7) | <0.001 | 0.115 |
| D1 | 0.929 (0.884-0.973) | 21.4 | 84.2 (75.8-90.0) | 90.0 (74.4-96.5) | <0.001 | 0.009 |
| D2 | 0.965 (0.936-0.994) | 32.3 | 88.7 (80.8-93.5) | 96.7 (83.3-99.8) | <0.001 | <0.001 |
| D3 | 0.979 (0.958-1.00) | 38.0 | 88.4 (80.4-93.4) | 100.0 (88.6-100.0) | <0.001 | <0.001 |
| D5-7 | 0.994 (0.986-1.00) | 36.5 | 95.5 (89.0-98.2) | 100.0 (88.6-100.0) | <0.001 | 0.024 |

Results derive from receiver operating characteristic (ROC) analysis. Best cutoff value was calculated by maximizing the Youden index. *Adjusted p-value derive from multivariable generalized linear regression models (GLMs) after adjustment for age, sex and renal function (estimated glomerular filtration rate).

**Abbreviations.** AIS: acute ischemic stroke; AUC: area under the curve; PLR: positive likelihood ratio, sGFAP: serum glial fibrillary acidic protein.

**Supplemental Table S2.** Spearman’s correlations of AIS patients of the Halle cohort (n=102).

|  | **sGFAP D1** | **sGFAP D2** | **sGFAP D3** | **sGFAP D5-7** |
| --- | --- | --- | --- | --- |
| ASPECTS on admission | rho=-0.273  p=0.006  n=100 | rho=-0.449  p<0.001  n=95 | rho=-0.411  p<0.001  n=93 | rho=-0.465  p<0.001  n=88 |
| ASPECTS at 24-72h | rho=-0.316  p=0.002  n=94 | rho=-0.544  p<0.001  n=89 | rho=-0.527  p<0.001  n=87 | rho=-0.483  p<0.001  n=82 |
| NIHSS on admission | rho=0.545  p<0.001  n=102 | rho=0.463  p<0.001  n=97 | rho=0.547  p<0.001  n=95 | rho=0.483  p<0.001  n=89 |
| NIHSS at 24h | rho=0.587  p<0.001  n=102 | rho=0.607  p<0.001  n=97 | rho=0.632  p<0.001  n=95 | rho=0.714  p<0.001  n=89 |
| NIHSS at 48h | rho=0.530  p<0.001  n=101 | rho=0.582  p<0.001  n=97 | rho=0.636  p<0.001  n=95 | rho=0.685  p<0.001  n=89 |
| NIHSS at 72h | rho=0.509  p<0.001  n=100 | rho=0.582  p<0.001  n=97 | rho=0.636  p<0.001  n=95 | rho=0.685  p<0.001  n=89 |
| NIHSS at discharge | rho=0.417  p<0.001  n=85 | rho=0.379  p<0.001  n=84 | rho=0.397  p<0.001  n=83 | rho=0.506  p<0.001  n=78 |
| eGFR | - | - | rho=-0.229  p=0.026  n=95 | rho=-0.266  p=0.013  n=89 |
| hemoglobin (mmol/l) | rho=-0.378  p<0.001  n=102 | rho=-0.230  p=0.023  n=97 | rho=-0.219  p=0.033  n=95 | rho=-0.224  p=0.035  n=89 |
| sGFAP D1 | 1 | rho=0.726  p<0.001  n=97 | rho=0.732  p<0.001  n=95 | rho=0.680  p<0.001  n=89 |
| sGFAP D2 | rho=0.726  p<0.001  n=97 | 1 | rho=0.904  p<0.001  n=95 | rho=0.808  p<0.001  n=88 |
| sGFAP D3 | rho=0.732  p<0.001  n=96 | rho=0.904  p<0.001  n=95 | 1 | rho=0.875  p<0.001  n=88 |
| sGFAP D5-7 | rho=0.680  p<0.001  n=89 | rho=0.808  p<0.001  n=88 | rho=0.875  p<0.001  n=88 | 1 |
| sNfL D1 | rho=0.471  p<0.001  n=101 | rho=0.399  p<0.001  n=97 | rho=0.314  p=0.002  n=95 | rho=0.416  p=0.014  n=89 |
| sNfL D2 | rho=516  p<0.001  n=97 | rho=0.546  p<0.001  n=97 | rho=0.464  p<0.001  n=95 | rho=0.580  p<0.001  n=88 |
| sNfL D3 | rho=546  p<0.001  n=95 | rho=0.580  p<0.001  n=95 | rho=0.558  p<0.001  n=95 | rho=0.688  p<0.001  n=88 |
| sNfL D5-7 | rho=0.594  p<0.001  n=89 | rho=0.618  p<0.001  n=88 | rho=0.645  p<0.001  n=88 | rho=0.699  p<0.001  n=89 |

Data are reported as Spearman’s rho (p-value) [n. observations] if statistically significant. sGFAP concentrations are calculated in ng/ml. Correlations of sGFAP with age, BMI, triglycerides, total cholesterol, HDL, LDL, INR, troponin T, and NT-pro-BNP are not displayed because not significant at any timepoint. Correlations between sGFAP and sNfL were still significant after correction for eGFR at multivariable linear regression except for sNfL D1 with sGFAP D1 (n=125, p=0.176).

**Abbreviations.** AIS: acute ischemic stroke; ASPECTS: Alberta Stroke Program CT Score; BMI: body mass index; eGFR: estimated glomerular filtration rate; HDL: high-density lipoprotein; INR: international normalized ratio; LDL: low-density lipoprotein; NIHSS: National Institute of Health Stroke Scale; NT-pro-BNP: N-terminal prohormone of the brain natriuretic peptide; sGFAP: serum glial fibrillary acidic protein; sNfL: serum neurofilament light chain.

**Supplemental Table S3.** Spearman’s correlations of AIS patients of the Würzburg cohort (n=470).

|  | **sGFAP D1** | **sGFAP D2** | **sGFAP D3** | **sGFAP D5-7** |
| --- | --- | --- | --- | --- |
| ASPECTS on admission | rho=-0.385  p<0.001  n=118 | rho=-0.631  p<0.001  n=213 | rho=-0.669  p<0.001  n=123 | - |
| NIHSS on admission | rho=0.316  p<0.001  n=134 | rho=0.466  p<0.001  n=235 | rho=0.347  p<0.001  n=123 | rho=0.344  p=0.022  n=44 |
| NIHSS at 24h | rho=0.576  p<0.001  n=134 | rho=0.531  p<0.001  n=236 | rho=0.534  p<0.001  n=213 | rho=0.613  p<0.001  n=44 |
| NIHSS at 48h | rho=0.524  p<0.001  n=132 | rho=0.558  p<0.001  n=215 | rho=0.586  p<0.001  n=123 | rho=0.703  p<0.001  n=44 |
| NIHSS at 72h | rho=0.486  p<0.001  n=129 | rho=0.540  p<0.001  n=203 | rho=0.529  p<0.001  n=122 | rho=0.696  p<0.001  n=44 |
| NIHSS at discharge | rho=0.449  p<0.001  n=102 | rho=0.555  p<0.001  n=172 | rho=0.433  p<0.001  n=98 | rho=0.607  p<0.001  n=36 |
| sGFAP D1 | 1 | - | rho=0.653  p=0.001  n=22 | - |
| sGFAP D2 | - | 1 | rho=0.923  p<0.001  n=19 | rho=0.846  p=0.001  n=12 |
| sGFAP D3 | rho=0.653  p=0.001  n=22 | rho=0.923  p<0.001  n=19 | 1 | rho=0.705  p<0.001  n=26 |
| sGFAP D5-7 | - | rho=0.846  p=0.001  n=12 | rho=0.705  p<0.001  n=26 | 1 |
| sNfL D1 | rho=0.511  p<0.001  n=134 | rho=0.800  p=0.333  n=4 | rho=0.054  p=0.813  n=22 | rho=0.069  p=0.795  n=17 |
| sNfL D2 | rho=0.800  p=0.333  n=4 | rho=0.406  p<0.001  n=236 | rho=0.546  p=0.019  n=19 | rho=0.503  p=0.099  n=12 |
| sNfL D3 | rho=0.475  p=0.019  n=24 | rho=0.691  p=0.001  n=19 | rho=0.426  p<0.001  n=123 | rho=0.445  p=0.023  n=26 |
| sNfL D5-7 | rho=0.414  p=0.078  n=19 | rho=0.413  p=0.0184  n=12 | rho=0.433  p=0.024  n=27 | rho=0.554  p<0.001  n=44 |

Data are reported as Spearman’s rho (p-value) [n. observations] if statistically significant. sGFAP concentrations are calculated in ng/ml. Correlations of sGFAP with age and eGFR are not displayed because not significant at any timepoint. Correlations between sGFAP and sNfL were still significant after correction for eGFR at multivariable linear regression except for:

- sNfL D1 with sGFAP D3 (n=22, p=0.911) and D5-7 (n=17, p=0.058);

- sNfL D2 with sGFAP D1 (n=4, p=0.108), D3 (n=19, 0.588) and D5-7 (n=12, p=0.589);

- sNfL D5-7 with sGFAP D2 (n=12, p=0.136) and D3 (n=27, p=0.199).

**Abbreviations.** AIS: acute ischemic stroke; ASPECTS: Alberta Stroke Program CT Score; eGFR: estimated glomerular filtration rate; NIHSS: National Institute of Health Stroke Scale; sGFAP: serum glial fibrillary acidic protein.

**Supplemental Table S4.** Frequency of HT in AIS patients.

|  | **Halle cohort (n=102)** | **Würzburg cohort (n=464)** |
| --- | --- | --- |
| all HT | 26 (25.5) | 249 (53.7) |
| Intracerebral hemorrhage (ICH) | 22 (21.6) | 221 (47.6) |
| HT 1a (HI1) | - | 74 (15.9) |
| HT 1b (HI2) | - | 70 (15.1) |
| HT 1c (PH1) | - | 47 (10.1) |
| HT 2 (PH2) | - | 18 (3.9) |
| HT 3a | - | 12 (2.6) |
| Intracranial extra-cerebral hemorrhage | - | 103 (22.2) |
| HT 3b (IVH) | - | 22 (4.7) |
| HT 3c (SAH) | 4 (3.9) | 75 (16.2) |
| HT 3d (SDH) | - | 6 (1.3) |

HT types are reported as n. of cases (%) according to the Heidelberg bleeding classification (ref. 13), namely:

- HT 1a (HI1): Scattered small petechiae within the infarcted tissue without mass effect;

- HT 1b (HI2): confluent petechiae within the infarcted issue without mass effect;

- HT 1c (PH1): hematoma within infarcted tissue, occupying <30%, without substantive mass effect;

- HT 2 (PH2): hematoma occupying 30% or more of the infarcted tissue, with obvious mass effect;

- HT 3a: parenchymal hematoma (PH) remote from infarcted brain tissue;

- HT 3b: intraventricular hemorrhage (IVH);

- HT 3c: subarachnoid hemorrhage (SAH);

- HT 3d: subdural hemorrhage (SDH).

Complete data on HT subtype were available only for the Würzburg cohort, whereas we have only data on ICH and SAH for patients of the Halle cohort. In the Würzburg, sum of cases with intra- and extra-cerebral hemorrhage exceeds total n. of cases because patients could have more than one type of HT

**Abbreviations.** AIS: acute ischemic stroke; HI: hemorrhagic infarction; HT: hemorrhagic transformation; ICH: intracerebral hemorrhage; IVH: intraventricular hemorrhage; PH: parenchymal hematoma; SAH: subarachnoid hemorrhage; SDH: subdural hemorrhage.

**Supplemental Table S5.** Correlations between neuronal/astroglial markers and inflammation parameters.

|  | **sGFAP D1** | **sGFAP D2** | **sGFAP D3** | **sGFAP D5-7** |
| --- | --- | --- | --- | --- |
| CRP D1 | 0.078 (ns) | 0.065 (ns) | 0.020 (ns) | 0.048 (ns) |
| CRP D2 | 0.242* | 0.305* | 0.326** | 0.302* |
| CRP D3 | 0.303* | 0.428*** | 0.474*** | 0.511*** |
| CRP D5-7 | 0.361** | 0.482*** | 0.515*** | 0.450*** |
| leucocyte count D1 | 0.250* | 0.323** | 0.309** | 0.335** |
| leucocyte count D2 | 0.343** | 0.490** | 0.417*** | 0.342** |
| leucocyte count D3 | 0.216 (ns) | 0.329** | 0.348** | 0.338** |
| leucocyte count D5-7 | 0.312** | 0.318** | 0.316** | 0.318* |
|  | **sNfL D1** | **sNfL D2** | **sNfL D3** | **sNfL D5-7** |
| CRP D1 | 0.406*** | 0.368** | 0.328** | 0.172 (ns) |
| CRP D2 | 0.286* | 0.330** | 0.432*** | 0.400** |
| CRP D3 | 0.287* | 0.343** | 0.549*** | 0.540*** |
| CRP D5-7 | 0.336** | 0.387** | 0.521*** | 0.445*** |
| leucocyte count D1 | 0.286 (ns) | 0.324** | 0.318** | 0.327** |
| leucocyte count D2 | 0.304** | 0.334** | 0.394** | 0.423*** |
| leucocyte count D3 | 0.316* | 0.336** | 0.431*** | 0.495*** |
| leucocyte count D5-7 | 0.367** | 0.388** | 0.339** | 0.295* |

CRP concentrations were considered in mg/l, leucocyte count in 10^3^ cells/μl, sGFAP concentrations in ng/ml and sNfL concentrations in pg/ml. Results are reported as Spearman’s rho coefficient and p-value (*<0.05; *<0.01; ***<0.001).

**Abbreviations.** CRP: C reactive protein; sGFAP: serum glial fibrillary acidic protein; sNfL: serum neurofilament light chain.

**Supplemental Table S6.** Prognostic value of sGFAP for clinical outcomes after AIS.

| **sGFAP** | **Halle cohort** | | | **Würzburg cohort** | | |
| --- | --- | --- | --- | --- | --- | --- |
| **3-month mRS of 3-6 vs. 0-2 or unchanged to pre-stroke mRS** | **OR (95%CI)** | **p** | **n. cases** | **OR (95%CI)** | **p** | **n. cases** |
| first available* | - | - | - | 1.015 (1.005-1.025) | 0.004 | 450 |
| D1 | 1.013 (0.987-1.039) | 0.322 | 102 | 1.012 (0.989-1.035) | 0.331 | 130 |
| D2 | 1.001 (0.9997-1.002) | 0.161 | 97 | 1.020 (1.003-1.037) | 0.021 | 226 |
| D3 | 1.005 (0.997-1.014) | 0.229 | 95 | 1.008 (0.996-1.019) | 0.192 | 119 |
| D5-7 | 1.012 (0.991-1.034) | 0.282 | 89 | 1.053 (0.958-1.16) | 0.284 | 44 |
| **Mortality at 3 months** | **OR (95%CI)** | **p** | **n. cases** | **OR (95%CI)** | **p** | **n. cases** |
| first available* | - | - | - | 1.006 (1.002-1.010) | 0.004 | 450 |
| D1 | 1.003 (0.999-1.007) | 0.141 | 102 | 1.012 (0.996-1.028) | 0.140 | 130 |
| D2 | 1.0003 (0.9998-1.001) | 0.228 | 97 | 1.005 (0.9998-1.011) | 0.058 | 226 |
| D3 | 1.0006 (0.99998-1.001) | 0.057 | 95 | 1.011 (1.001-1.020) | 0.025 | 119 |
| D5-7 | 1.0004 (0.9998-1.001) | 0.153 | 89 | 1.0004 (0.995-1.006) | 0.887 | 44 |
| **Intra-hospital mortality** | **OR (95%CI)** | **p** | **n. cases** | **OR (95%CI)** | **p** | **n. cases** |
| first available* | - | - | - | 1.005 (1.002-1.009) | 0.004 | 462 |
| D1 | 1.003 (0.999-1.007) | 0.115 | 102 | 1.015 (0.997-1.032) | 0.097 | 132 |
| D2 | 1.0004 (0.9999-1.001) | 0.098 | 97 | 1.003 (0.998-1.007) | 0.236 | 232 |
| D3 | 1.0006 (1.00001-1.001) | 0.047 | 95 | 1.013 (1.003-1.023) | 0.011 | 123 |
| D5-7 | 1.0005 (0.9999-1.001) | 0.116 | 89 | 1.001 (0.995-1.007) | 0.777 | 44 |

Results were obtained with GLMs after accounting for age, sex, eGFR and NIHSS on admission. *Given that time-to-sampling was different in patients of the Würzburg cohort, we also performed a sensitivity analysis in the full dataset by considering the first available sample and adjusting models also for time from onset to blood sampling in hours. Data on “first available” sGFAP for the Halle cohort were not reported given that it coincided with D1.

**Abbreviations.** AIS: acute ischemic stroke; eGFR: estimated glomerular filtration rate; NIHSS: National Institute of Health Stroke Scale; OR: odds ratio; sGFAP: serum glial fibrillary acidic protein.

**Supplemental Table S7.** Prognostic value of sNfL for clinical outcomes after AIS.

| **sNfL** | **Halle cohort** | | | **Würzburg cohort** | | |
| --- | --- | --- | --- | --- | --- | --- |
| **3-month mRS of 3-6 vs. 0-2 or unchanged to pre-stroke mRS** | **OR (95%CI)** | **p** | **n. cases** | **OR (95%CI)** | **p** | **n. cases** |
| first available* | - | - | - | 1.002 (1.001-1.003) | 0.007 | 456 |
| D1 | 1.003 (0.999-1.007) | 0.170 | 101 | 1.005 (1.0002-1.009) | 0.042 | 133 |
| D2 | 1.004 (0.9995-1.008) | 0.080 | 97 | 1.001 (0.9995-1.003) | 0.158 | 227 |
| D3 | 1.003 (1.0003-1.007) | 0.048 | 95 | 1.0008 (0.999-1.002) | 0.375 | 123 |
| D5-7 | 1.002 (1.0001-1.004) | 0.0499 | 89 | 1.002 (0.999-1.005) | 0.136 | 46 |
| **Mortality at 3 months** | **OR (95%CI)** | **p-value** | **n. cases** | **OR (95%CI)** | **p-value** | **n. cases** |
| first available* | - | - | - | 1.0007 (1.00003-1.001) | 0.041 | 456 |
| D1 | 1.003 (1.0003-1.005) | 0.025 | 101 | 1.002 (1.0003-1.004) | 0.025 | 133 |
| D2 | 1.004 (1.001-1.006) | 0.007 | 97 | 1.0001 (0.999-1.001) | 0.779 | 227 |
| D3 | 1.003 (1.001-1.005) | 0.004 | 95 | 1.002 (1.0004-1.004) | 0.018 | 123 |
| D5-7 | 1.0009 (0.9998-1.002) | 0.098 | 89 | 1.001 (0.9998-1.002) | 0.113 | 46 |
| **Intra-hospital mortality** | **OR (95%CI)** | **p-value** | **n. cases** | **OR (95%CI)** | **p-value** | **n. cases** |
| first available* | - | - | - | 1.001 (1.0006-1.002) | <0.001 | 469 |
| D1 | 1.002 (0.9999-1.004) | 0.065 | 101 | 1.002 (1.001-1.004) | 0.010 | 135 |
| D2 | 1.004 (1.001-1.007) | 0.010 | 97 | 1.0004 (0.9995-1.001) | 0.351 | 233 |
| D3 | 1.004 (1.001-1.006) | 0.004 | 95 | 1.002 (1.001-1.004) | 0.008 | 128 |
| D5-7 | 1.0009 (0.9997-1.002) | 0.127 | 89 | 1.001 (0.9998-1.003) | 0.078 | 46 |

Results were obtained with GLMs after accounting for age, sex, eGFR and NIHSS on admission. *Given that time-to-sampling was different in patients of the Würzburg cohort, we also performed a sensitivity analysis in the full dataset by considering the first available sample and adjusting models also for time from onset to blood sampling in hours. Data on “first available” sNfL for the Halle cohort were not reported given that it coincided with D1.

**Abbreviations.** AIS: acute ischemic stroke; eGFR: estimated glomerular filtration rate; NIHSS: National Institute of Health Stroke Scale; OR: odds ratio; sNfL: serum neurofilament light chain protein.
